# White Spot Syndrome Virus Orf514 Encodes a *Bona Fide* DNA Polymerase

**DOI:** 10.3390/molecules16010532

**Published:** 2011-01-12

**Authors:** Enrique de-la-Re-Vega, Karina D. Garcia-Orozco, Aldo A. Arvizu-Flores, Gloria Yepiz-Plascencia, Adriana Muhlia-Almazan, Jesús Hernández, Luis G. Brieba, Rogerio R. Sotelo-Mundo

**Affiliations:** 1 Centro de Investigación en Alimentación y Desarrollo, A.C. (CIAD), Carretera a Ejido La Victoria Km 0.6, Apartado Postal 1735, Hermosillo, Sonora 83000, Mexico; 2 Departamento de Ciencias Químico Biológicas, Universidad de Sonora, Blvd. Luis Encinas y Rosales S/N, Col. Centro, Hermosillo, Sonora 83000, Mexico; 3 Laboratorio Nacional de Genómica para la Biodiversidad (LANGEBIO), Centro de Investigación y EstudiosAvanzados (CINVESTAV Campus Guanajuato), Km 9.6 Libramiento Norte Carretera Irapuato-León, Apartado Postal 629, Irapuato, Guanajuato 36500, Mexico

**Keywords:** DNA polymerase family B, WSSV, white spot syndrome virus, polymerase activity, Crustacea, shrimp, prawn, *Litopenaeus vannamei*, Decapoda, invertebrate

## Abstract

White spot syndrome virus (WSSV) is the causative agent of white spot syndrome, one of the most devastating diseases in shrimp aquaculture. The genome of WSSV includes a gene that encodes a putative family B DNA polymerase (ORF514), which is 16% identical in amino acid sequence to the Herpes virus 1 DNA polymerase. The aim of this work was to demonstrate the activity of the WSSV ORF514-encoded protein as a DNA polymerase and hence a putative antiviral target. A 3.5 kbp fragment encoding the conserved polymerase and exonuclease domains of ORF514 was overexpressed in bacteria. The recombinant protein showed polymerase activity but with very low level of processivity. Molecular modeling of the catalytic protein core encoded in ORF514 revealed a canonical polymerase fold. Amino acid sequence alignments of ORF514 indicate the presence of a putative PIP box, suggesting that the encoded putative DNA polymerase may use a host processivity factor for optimal activity. We postulate that WSSV ORF514 encodes a *bona fide* DNA polymerase that requires accessory proteins for activity and maybe target for drugs or compounds that inhibit viral DNA replication.

## 1. Introduction

The white spot syndrome virus (WSSV) is the causative agent of the white spot syndrome, which is the most serious viral disease of cultured shrimp in the world [1]. Since 1993, this virus has been found in most of the shrimp farming regions in South Asia, Europe, Australia and Latin America. WSSV is highly lethal and represents the most important threat for shrimp aquaculture industry [2]. The WSSV genome is completely sequenced and it contains ORFs corresponding to enzymes involved in nucleic acid metabolism [3]. The WSSV enzymes involved in DNA replication and characterized to date are ribonucleotide reductase (RR) [4], dUTPase, a chimeric thymidine kinase-thymidylate kinase (TK-TMK) [4], and thymidylate synthase [5]. The WSSV ORF514 (in the early literature, ORF514 is also known as ORF27, both corresponding to a putative WSSV DNA polymerase) is 7,056 nucleotides long and encodes for a 2,351 amino acids polypeptide (262 kDa), that is classified as a putative family B DNA polymerase [6]. Although the ORF514 amino acid sequence contains only 16% identity to the herpes simplex virus DNA polymerase 1 (HSV) amino acid sequence. The polymerase (*pol*) and exonuclease (*exo*) domains of family B DNA polymerases are easily identified using bioinformatic tools. 

Expression of ORF514 has been detected very rapidly after WSSV virus infection, since the viral transcript is present as early as 2 h after viral infection [6]. However, the biochemical function of the gene product encoded in ORF514 has not been experimentally demonstrated yet. Thus, the aim of this work was to produce and purify the recombinant protein encoded by the WSSV ORF514 and to evaluate its polymerase activity. Since family B DNA polymerases are drug targets, ORF514 may be a potential target for antiviral therapy against WSSV.

## 2. Results and Discussion

### 2.1. WSSV ORF514 Contains the Functional Domains of a Family B DNA Polymerase

**Figure 1:**
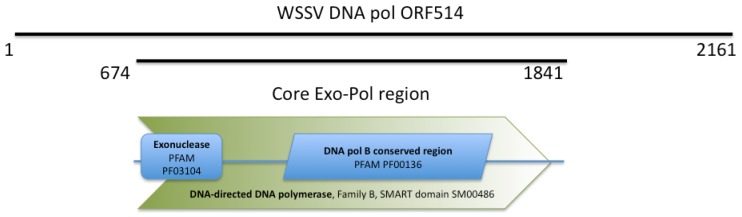
Domain structure of the core region of the WSSV ORF514. The domains identified in the sequence include the reference number either to the PFAM (http://pfam.sanger.ac.uk) or SMART (http://smart.embl-heidelberg.de) domain.

WSSV ORF514 encodes a putative protein of 261 kDa that belongs to the family B DNA polymerases. ORF514 is unusually long. This unusual length is due to non-conserved additional sequences in its amino and carboxy terminal ends. The central region of the ORF514, from residue 674 to 177, encodes a putative DNA polymerase with the conserved *exo* and *pol* DNA polymerase family B domains (Figure 1).

**Figure 2:**
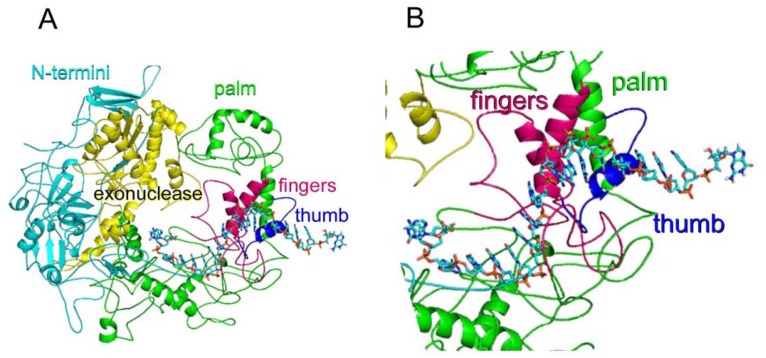
Molecular model of the core region of WSSV ORF514. **(A)** The sub domains were colored as follows: finger in magenta, thumbs in blue, palm in green. The domains were colored as follows: exonuclease in yellow, N-termini in cyan; **(B)** Close up to the active site where the fingers, palm and thumb are depicted.

To illustrate if the selected core region contained functional domains of family B DNA polymerases, the amino acid sequence of the WSSV ORF514 core region was modeled on a closed DNA-bound conformation using the HSV 1 DNA polymerase crystal structure as a template (PDB: 2GV9). The *pol* and *exo* domains were identified in the theoretical model (Figure 2A).

The *pol* domain (Figure 2B) is divided in three sub domains: the fingers (magenta), that interact with the incoming nucleoside triphosphate and the template; the thumb (blue), which is involved in positioning the DNA duplex, processivity and translocation; and the palm (green), that is involved in the phosphoryl transfer reaction [7,8]. The *exo* domain (yellow) is responsible for replication fidelity. The model fitted the classical hand shape of a DNA polymerase: a “right hand holding the DNA”. Several conserved basic residues were identified in the finger subdomain at the active site in contact with the phosphate moieties of DNA template. The acidic residues, which are required for catalysis of the incoming triphosphate, at the palm subdomain were also identified in the structural model, suggesting that our model is in good agreement with the topological structure of DNA polymerases. The DNA *pol* encoded by ORF514 in WSSV genome has several insertions with respect to other DNA polymerases known to date. These sequences are found mainly at loop regions of the theoretical model and many of them adopted secondary structure elements in a consistently form within several independent models.

### 2.2. WSSV ORF514 has Polymerase Activity

**Figure 3:**
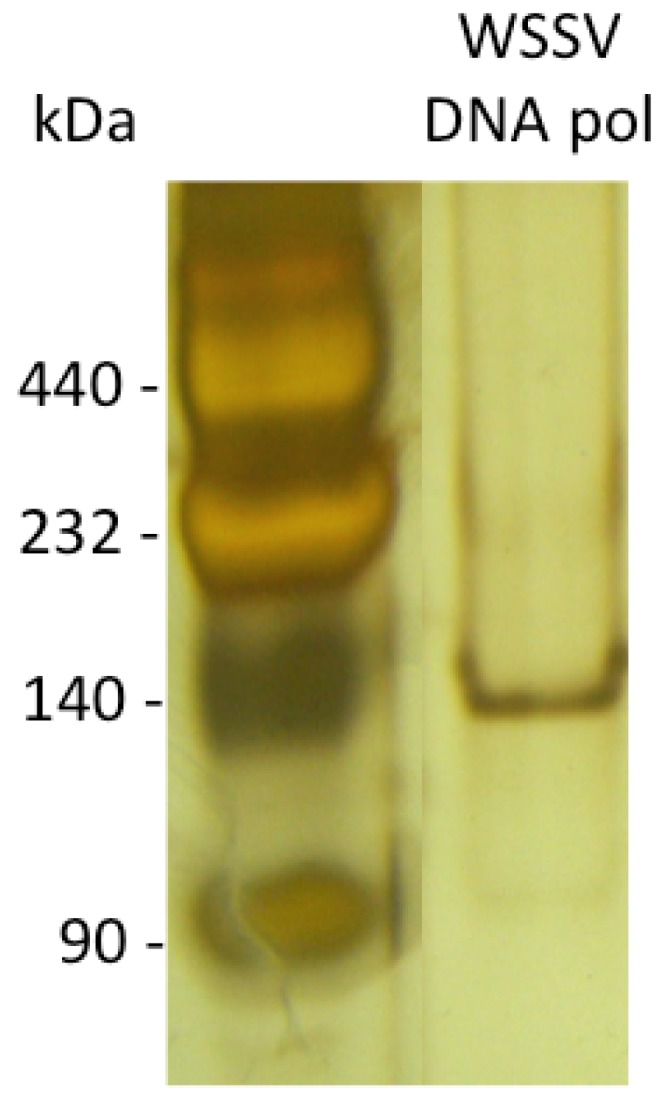
Purification of WSSV DNA pol. Native silver-stained gel of purified WSSV DNA *pol*.

This core domain was selected for recombinant expression, since other viral DNA polymerases are functional having only the *pol* and *exo* domains. For the biochemical characterization of the WSSV ORF514-encoded protein, the core region was produced as a recombinant protein in bacteria. Recombinant expression of the ORF514 viral coding region at 16 °C led to very low yields of a soluble protein (data not shown). To increase yields, a synthetic gene with optimized codons was synthesized for expression in bacteria. We obtained low yields but enough for biochemical characterization (100 μg of purified protein per 1 L bacterial culture). The WSSV DNA polymerase encoded in ORF514 was detected as a 140 kDa polypeptide in native polyacrylamide electrophoresis (Figure 3) and its polymerase activity was shown.

**Figure 4:**
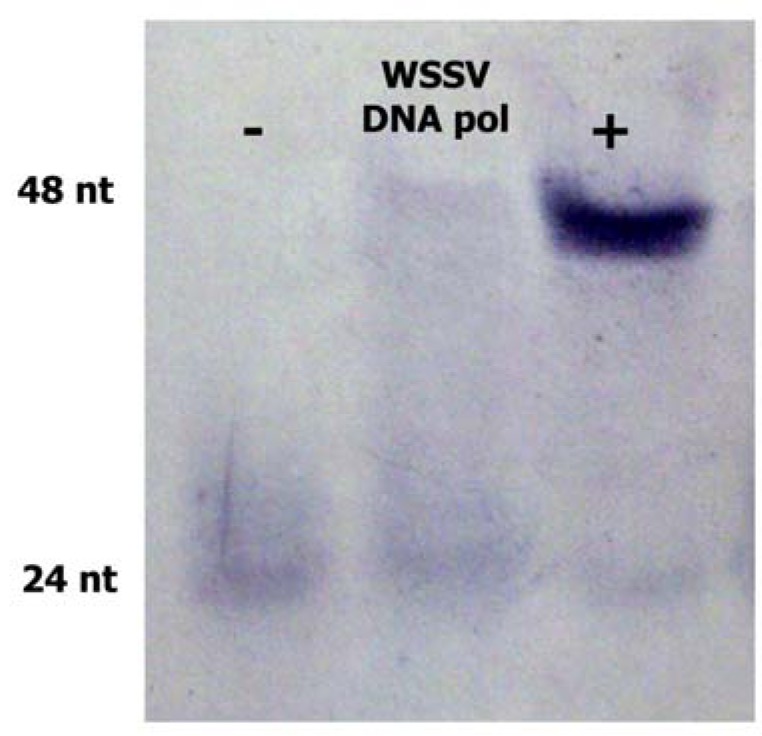
Polymerase activity of the recombinant WSSV DNA pol. WSSV DNA *pol* was tested in a nucleotide incorporation assay (central lane). The Klenow fragment was the positive control (lane +). A reaction without polymerase was ran as negative control (lane).

The recombinant WSSV core ORF514 protein was tested for DNA polymerase activity (Figure 4) with the non-radioactive polymerase activity assay. Under the experimental condition, recombinant WSSV incorporated 24 nt in 16 min, similarly to the control DNA polymerase (Klenow fragment) that incorporated the 24 nt in 2 min (lane +) but which much more efficiency. These results indicate that ORF514 encodes a *bona fide* viral DNA polymerase, and the low rate of incorporation observed *in vitro* suggests that this polymerase may require accessory proteins for enhanced processivity.

The DNA polymerase processivity factor or sliding clamp in known replication systems allows the core polymerase to remain associated to the DNA template and increase the rate of nucleotide incorporation [9]. The HSV UL42 protein increases the processivity and elongation rate of the herpes DNA polymerase, and requires a specific amino acid region in the DNA polymerase for protein-protein interactions [9,10,11]. Also, complementation of the DNA polymerase of *Archaeoglobus fulgidus* with clamp/clamp replication factor C and proliferating cell nuclear antigen (RFC/PCNA) is needed to increase the processivity of the archeal polymerase [12].

### 2.3. Interaction with Host Replication Factors

**Figure 5:**
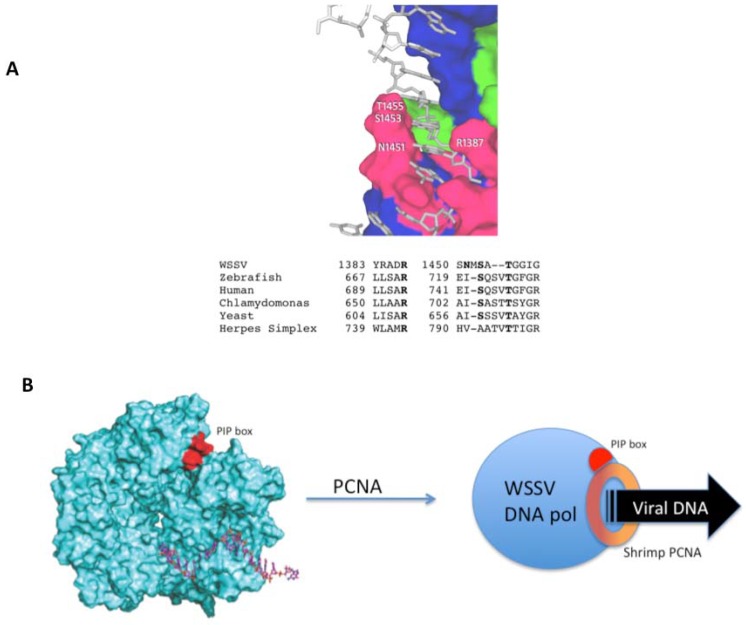
A proposal for a processive complex for WSSV viral replication. (**A**) Conserved residues of the finger domain are depicted, and sequence alignment shows the degree of sequence conservation. The sequences correspond to the following GenBank accession numbers: WSSV: AAK77696ORF27; Zebrafish: *Danio rerio* AAI63875; Human ABB29977; *Chlamydomonas reinhardtii* XP_001689909. For yeast and herpes the sequences were obtained from the Protein Data Base: Yeast: *Saccharomyces cerevisiae* PDB3IAY; Herpes Simplex Virus Type 1: 2GV9; (**B**) To the left, the molecular model of WSSV core DNA pol, showing bound DNA and the PIP box, a site where PCNA binds.

Structural features found in the WSSV DNA polymerase molecular model include invariant residues in the fingers subdomain (Figure 5a). Although the overall identity with the HSV DNA pol is 16%, the finger region contains invariant residues R1387, S1453 and T1455 from humans to yeast and HSV. The finger subdomain appears as the element that holds the double helix DNA during replication. Although the homology model is constrained to the template conformation, is remarkable that polar and basic residues are in position to have adequate interactions with DNA, as found in the template HSV 1 DNA *pol*.

Since many polymerases are known to require processivity factors, analysis of the core WSSV DNA polymerase for possible binding sites to these proteins was done. A PIP box [13] was identified within the linear amino acid sequence and also in the structural model (Figure 5b). PIP box appears in the accessible surface of the model (in red), predicting that such host factors may bind and increase nucleotide incorporation rates.

The PIP box domain consists of the sequence QxxHxxA, were “x” is any amino acid, “H” represents a hydrophobic residue and “A” represents an aromatic residue [13,14] (Figure 5b). The PIP box, which is a short amino acid sequence found in many polymerases, allows interaction between the DNA polymerase and PCNA (Proliferating Cell Nuclear Antigen) to form the replication complex. DNA polymerase performs a processive DNA synthesis only when bound to PCNA among other processivity factors [14]. The WSSV ORF514 amino acid sequence contains the sequence ^680^QHKILYY^686^, which corresponds to the PIP box, hence, is likely that the viral DNA polymerase uses the host cell processivity factor. An analogous situation is present in T7 DNA replication in which T7 DNA polymerase uses the host *E. coli* thioredoxin as processivity factor. [15] Recently, the PCNA from the shrimp has been identified [16], confirming the presence of this accessory replication factor in the shrimp genome. Although the WSSV DNA pol was modeled using an experimental crystallographic model, the fact that the PIP box is found in the surface of the polymerase model suggests that shrimp PCNA may bind. Further experimental work is required to test this hypothesis.

Considering the limited resolution of protein modeling is remarkable that the residues adopted the cognate finger conformation; nonetheless, a crystal structure of this protein in complex with DNA would be the way forward to further understand the actual mechanism of WSSV viral DNA replication.

## 3. Experimental 

### 3.1. Materials

*Escherichia coli* strain TOP10 (Stratagene, La Jolla CA, USA) was used for DNA cloning. *E. coli* BL21 Rosetta Novagen (San Diego CA, USA), was used for the over expression of the 3.5 Kb ORF514. Synthetic gene services and vector pJexpress401 were from DNA 2.0 (Menlo Park, CA, USA). Chromatography columns were from GE Healthcare (Piscataway, NJ, USA), labeled primers were from IDT (Coralville, IA, USA).

### 3.2. WSSV ORF514 Expression Construct

The WSSV ORF514 coding region (GenBank AAK77696.1) containing the putative polymerase and exonuclease domains was used for the design of the synthetic gene. The synthetic amino acid terminal sequence started with a methionine residue followed by a six-histidine residues tag and by the fragment of the ORF514 (from residues M674 to V1841), which contain the polymerase and exonuclease domains. The nucleotide sequence of the construct was optimized for its expression in *E. coli* [17], synthesized and cloned in-frame within the expression vector pJexpress401 by DNA 2.0 (Menlo Park, CA, USA). This vector contains the T7 promoter for functional expression in the *E. coli* BL21(DE3) strain.

### 3.3. Recombinant Expression of WSSV ORF514

The pJexpress401-WSSVORF514 synthetic construct was used to transform BL21(DE3) Rosetta *E. coli* cells. Cells were grown in 1 L TB media at 37 °C to an optical density of 0.5–0.6 (OD_500_), and IPTG was added at a final concentration of 0.1 mM. Recombinant expression of WSSV DNA pol was allowed for 4 h post-induction. From this point all subsequent steps were carried out at 4 °C. Cells were harvested by centrifugation at 5000 × *g* for 15 min, washed with 0.1% saline and then sonicated in 25 mM glycine, 1 mM PMSF, 0.1 mM EDTA, 1 mM DTT, pH 9.0 at a ratio of 8 mL per g pellet in ice bath. The soluble protein was recuperated by centrifugation at 10,000 × *g* for 20 min. The supernatant containing the expressed WSSV ORF514 was treated with streptomycin solution (5%) in a ratio of 15 mL per 100 mL of supernatant and recuperated by centrifugation at 10,000 *× g* for 20 min. Then, the recombinant protein encoded in WSSV ORF514 was precipitated with ammonium sulfate (40%). The pellet was then dissolved and dialyzed overnight against buffer C (25 mM glycine, 0.1 mM EDTA, 0.1 mM PMSF, 1 mM DTT, pH 9.0).

### 3.4. WSSV ORF514 Protein Purification

The recombinant protein was not amenable for purification using IMAC by the poly-histidine tag (data not shown). Therefore, a traditional purification scheme was applied, using anionic exchange chromatography with a DEAE FF HiTrap column (GE Healthcare, Piscataway, NJ, USA). Elution of the WSSV DNA pol was done with a linear gradient of NaCl to 1 M in running buffer C. A second step of chromatographic purification was done using phosphocelullose (Whatman GE Healthcare, Piscataway, NJ, USA), equilibrated with buffer C and eluted with buffer C plus 100 mM NaCl. A third step of purification was done using gel filtration chromatography in a 10 × 300 mm Biogel P150 column (Bio-Rad, Hercules, CA, USA) with buffer C plus 200 mM NaCl. Fractions containing the WSSV ORF514 encoded protein were concentrated using a Centricon Plus PL-30 (Millipore, Billerica, MA, USA). Fractions were analyzed by SDS-PAGE (8%) and stained with Coomassie Blue R-250.

### 3.5. DNA Polymerase Activity Assay

A nucleotide incorporation assay was used to demonstrate the DNA polymerase activity of the recombinant protein encoded in ORF514. A 24-mer primer (oligo A; 5’-CGCAGCCCACCTGCCCA- CCTAACT-3’) was 5’-labeled with digoxigenin (DIG) and hybridized to a template strand (oligo B; 5’-CCTTGGCACTAGCGCAGGGCAAGTTAGGTGGGCAGGTGGGCTGCG-3’. This is a non-isotopic implementation of the radioactive polymerase assay previously reported [18]. Since oligo A was synthesized with the DIG label, no labeling reaction was required. The hybridization of the DIG-oligo A with oligo B was done by mixing 2.2 µL and 3.2 µL of oligo A and oligo B (3 nM solutions) in a final volume of 100 µL of water. The reaction was incubated at 95 °C for 5 min and slowly cooled down for 2 h at room temperature. The activity reaction contained 2.8 pM of the hybridized template, 25 µM each dNTP, 0.176 nM of WSSV DNA pol in a reaction buffer of50 mM NaCl, 10 mM Tris-HCl, 10 mM MgCl_2_, 1 mM DTT, pH 7.5; the DNA polymerase I (Klenow fragment) was used as a positive control reaction. The reactions were incubated at 37 °C for 8–16 min and terminated by adding an equal volume of formamide stop solution (95 % formamide, 25 mM EDTA, 0.1% xylene cyanol, 0.01% bromophenol blue). The DNA products were separated on 18% denaturing polyacrylamide gels, containing 8 M urea. To the detect the DNA products labeled with DIG, the gels were transferred to a Hybond membrane using a semi-dry blotter (Bio-Rad) at 25 volts for 30 min, using 0.5 × TBE buffer to wet the membrane. The transferred DNA was fixed to the membrane by crosslinking with a mini UV oven for 30 s at 12,000 µJ. The membrane was rinsed with washing buffer (0.1 M maleic acid, 0.15 M NaCl, 0.3% Tween 20, pH 7.5) and blocked with blocking solution (Roche blocking reagent), for 30 min. Then, the membrane was incubated for 1 h with alkaline phophatase labeled anti-DIG diluted 1:20,000 in blocking solution, and then washed twice with buffer for 15 min. Color detection was done with NBT/BCIP (Nitro Blue tetrazolium chloride/5-Bromo-4-chloro-3-indolyl phosphate) according to the manufacturer (Bio-Rad).

### 3.6. WSSV DNA ORF514 Encoded DNA Polymerase Molecular Modeling

The homology modeling of the WSSV DNA polymerase encoded in ORF514 was done by superimposing the deduced core *exo* and *pol* domains in ORF514 amino acid sequence into the known crystallographic structure of the Herpes virus family B DNA polymerase type 1 (PDB: 2GV9) [19] using MOE 2009.10 (ChemComp, Montreal, Canada). The coordinates of DNA template and primer in complex with the enzyme were taken from the crystallographic structure of phage RB69 DNA pol replicating complex (PDB: 1IG9) [20] and included in the construction of the WSSV DNA pol model. The assignment of the WSSV DNA polymerase domains and figures of the resulting structure were drawn with PyMOL 1.0 [21].

## 4. Conclusions 

WSSV ORF514 encodes an enzyme with *exo* and *pol* activities, and low rates of DNA incorporation suggests that a sliding clamp complex is needed to keep the DNA polymerase associated to the template during viral DNA synthesis. In this work, the characterization of the core region of the ORF containing the catalytic domains is presented. Moreover, the localization of the PIP box into the surface of the WSSV DNA polymerase structural model suggests the recruitment of shrimp replication factors. 

Although the role of a DNA polymerase in viral replication is evident, two results must be presented in perspective. In one work the WSSV putative DNA polymerase was silenced using RNA interference [22]. In this case at days post-challenge, the relative survival rates of shrimp injected with dsRNA-*dna pol* was 50%. A cause for this result may be related to viral mRNA stability and protein stability, in which elimination of message does not abrogate viral DNA synthesis. Also, the expression profile has been studied, and DNA polymerase is expressed 6 h to 60 h post-infection [6]. Availability of the recombinant protein will allow production of antibodies to follow protein and mRNA levels and to further understand DNA polymerase dynamics. 

Another issue critical to DNA replication is the availability of deoxinucleotides. *De novo* nucleotide synthesis is represented by thymidylate synthase, which is present in shrimp and WSSV [5]. The other pathway involves nucleotide salvage, where thymidine kinase is the key enzyme and is also present in WSSV and very likely also in shrimp genome [23]. Therefore, in the absence of stable crustacean cell lines, a biochemical approach for understanding WSSV replication may be the way to follow toward the development of antiviral therapies for this emerging disease. 

## Abbreviations

ORF514, ORF27, open reading frame; WSSV DNA pol, white spot syndrome virus DNA polymerase; PMSF, phenyl methylsulphonyl fluoride; IPTG isopropyl-β-D-thiogalactopyranoside; DTT, dithiotreitol
